# Stabilin-1 is expressed in human breast cancer and supports tumor growth in mammary adenocarcinoma mouse model

**DOI:** 10.18632/oncotarget.8857

**Published:** 2016-04-20

**Authors:** Vladimir Riabov, Shuiping Yin, Bin Song, Aida Avdic, Kai Schledzewski, Ilja Ovsiy, Alexei Gratchev, Maria Llopis Verdiell, Carsten Sticht, Christina Schmuttermaier, Hiltrud Schönhaber, Christel Weiss, Alan P. Fields, Katja Simon-Keller, Frederick Pfister, Sebastian Berlit, Alexander Marx, Bernd Arnold, Sergij Goerdt, Julia Kzhyshkowska

**Affiliations:** ^1^ Institute for Transfusion Medicine and Immunology, Medical Faculty Mannheim, Ruprecht-Karls University of Heidelberg, Mannheim, Germany; ^2^ Department of Dermatology, Medical Faculty Mannheim, Ruprecht-Karls University of Heidelberg, Mannheim, Germany; ^3^ Institute of Pathology, Medical Faculty Mannheim, Ruprecht-Karls University of Heidelberg, Mannheim, Germany; ^4^ Center for Medical Research, Medical Faculty Mannheim, Ruprecht-Karls University of Heidelberg, Mannheim, Germany; ^5^ Department for Medical Statistics and Biomathematics, Medical Faculty Mannheim, Ruprecht-Karls University of Heidelberg, Mannheim, Germany; ^6^ Department of Cancer Biology, Mayo Clinic Comprehensive Cancer Center, Jacksonville, Florida, USA; ^7^ Department of Obstetrics and Gynaecology, University Medical Center Mannheim, Heidelberg University, Mannheim, Germany; ^8^ Department of Molecular Immunology, German Cancer Research Center, Heidelberg, Germany; ^9^ Laboratory for Translational Cellular and Molecular Biomedicine, Tomsk State University, Tomsk, Russia

**Keywords:** tumor-associated macrophages, scavenger receptor, SPARC, stabilin-1, breast cancer

## Abstract

Stabilin-1 is a multifunctional scavenger receptor expressed on alternatively-activated macrophages. Stabilin-1 mediates phagocytosis of “unwanted-self” components, intracellular sorting, and endocytic clearance of extracellular ligands including SPARC that modulates breast cancer growth. The expression of stabilin-1 was found on tumor-associated macrophages (TAM) in mouse and human cancers including melanoma, lymphoma, glioblastoma, and pancreatic insulinoma. Despite its tumor-promoting role in mouse models of melanoma and lymphoma the expression and functional role of stabilin-1 in breast cancer was unknown. Here, we demonstrate that stabilin-1 is expressed on TAM in human breast cancer, and its expression is most pronounced on stage I disease. Using stabilin-1 knockout (ko) mice we show that stabilin-1 facilitates growth of mouse TS/A mammary adenocarcinoma. Endocytosis assay on stabilin-1 ko TAM demonstrated impaired clearance of stabilin-1 ligands including SPARC that was capable of inducing cell death in TS/A cells. Affymetrix microarray analysis on purified TAM and reporter assays in stabilin-1 expressing cell lines demonstrated no influence of stabilin-1 expression on intracellular signalling. Our results suggest stabilin-1 mediated silent clearance of extracellular tumor growth-inhibiting factors (e.g. SPARC) as a mechanism of stabilin-1 induced tumor growth. Silent clearance function of stabilin-1 makes it an attractive candidate for delivery of immunomodulatory anti-cancer therapeutic drugs to TAM.

## INTRODUCTION

Multifunctional scavenger receptor stabilin-1 (STAB1, FEEL-1, CLEVER-1, KIAA0246) is expressed on resident tissue macrophages and sinusoidal endothelial cells in healthy organism, and its expression on both macrophages and different subtypes of endothelial cells is induced during chronic inflammation and tumor progression [1, 2].

Analysis of the stabilin-1 function in *in vitro* model systems revealed its involvement in endocytic and phagocytic clearance of “unwanted-self” components, intracellular sorting of endogenously synthesized chitinase-like protein SI-CLP, and transcytosis of growth hormone family member placental lactogen. Endocytic ligands of stabilin-1 include modified lipoproteins, apoptotic cells, cytokine GDF15/MIC1 and soluble component of extracellular matrix SPARC [3–6]. SPARC is secreted by multiple cell types, negatively regulates cell adhesion, exerts anti-proliferative and anti-angiogenic effects [7]. Several studies reported that SPARC inhibits primary growth of lung carcinoma, neuroblastoma and metastasis of breast cancer in animal tumor models [8–11]. Reduced SPARC expression was shown to correlate with poor prognosis in breast cancer patients [12]. Forced expression of SPARC in 4T1 breast cancer cell line resulted in reduced tumor growth *in vivo* [13]. SPARC was also found to inhibit proliferation of breast cancer cells *in vitro* [10]. Several studies demonstrated that SPARC induces apoptosis in ovarian, colon cancer, and neuroectodermal tumor cells either directly or in combination with other treatments [14–16]. Although stabilin-1 ligands such as SPARC are known to modulate tumor progression the role of endocytic clearance function of stabilin-1 in TAM and its relation to tumor growth was not studied before.

Stabilin-1, but not stabilin-2, is expressed on macrophages in humans and rodents, and is used as a specific marker for alternatively-activated macrophages *in vivo* [17, 18]. In human monocyte-derived macrophages and mouse bone marrow-derived macrophages stabilin-1 is induced by IL-4 in combination with dexamethasone or by dexamethasone alone (alternatively-activated macrophages) [19]. Stabilin-1 is expressed *in vivo* in tumor-associated macrophages (TAM) in mouse models of melanoma, pancreatic insulinoma and glioblastoma [20, 21]. We found that in murine B16F1 melanoma tumor model stabilin-1 is expressed on a subset of CD11b(+), F4/80(+) tissue macrophages predominantly on the tumor periphery, while only single stabilin-1+ macrophages have been detected inside tumor mass [21]. Recently, tumor-promoting role of stabilin-1was demonstrated in mouse models of B16 melanoma and EL-4 lymphoma [22].

The role of TAM in tumor progression was intensively studied in breast cancer models. It was established that in breast tumors TAM induce tumor growth and angiogenesis, migration and invasion of tumor cells, and participate in the formation of metastatic niches [23–25]. TAM support tumor growth by secretion of multiple growth factors stimulating neoplastic cell proliferation and induction of angiogenic switch [26–30]. Abundant expression of scavenger receptors by TAM including CD163, CD204 (MSR1) and CD206 (MMR, MRC1) is also widely reported [30–33]. The numbers of CD163 and CD204 expressing macrophages correlate with poor prognosis in several types of cancer [31, 33, 34]. However, functional role of macrophage scavenger receptors in induction of tumor growth is obscure.

The aim of our study was to examine the role of scavenger receptor stabilin-1 in the progression of breast cancer. By immunohistochemistry we found that stabilin-1 is expressed in a significant subset of TAM in patients with breast carcinoma, and its expression is higher during early stage of breast cancer progression (stage I) and in case of metastatic cancer (stage IV). Using stabilin-1 knockout mice we demonstrated that the absence of stabilin-1 on TAM results in reduced growth of TS/A mammary adenocarcinoma which is accompanied by impaired endocytic clearance function of TAM and reduced uptake of SPARC capable of inducing TS/A tumor cell death. We did not find any effect of stabilin-1 on transcriptional level suggesting that its silent clearance function is essential for the regulation of tumor growth.

## RESULTS

### Stabilin-1 is expressed on TAM in human breast cancer

**Figure 1:**
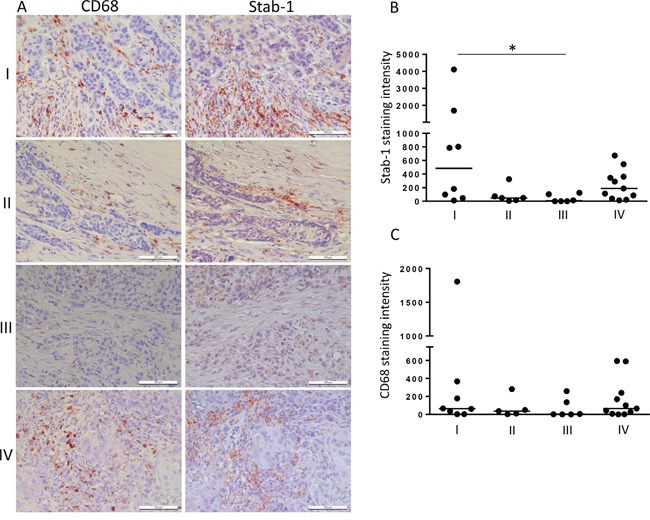
Stabilin-1 expression in human breast cancer Patient breast cancer samples were analysed by IHC for stabilin-1 (n=31) and CD68 (n=30) expression. **A.** Representative images from the same tumor regions are shown for TNM stages I, II, III and IV disease. Scale bars: 100 μm. Quantitative IHC analysis for the intensity of stabilin-1 **B.** and CD68 **C.** expression for TNM stages I, II, III and IV is shown as a fold change. *p<0.05, Kruskal-Wallis test with Dunn's multiple comparisons.

**Figure 2:**
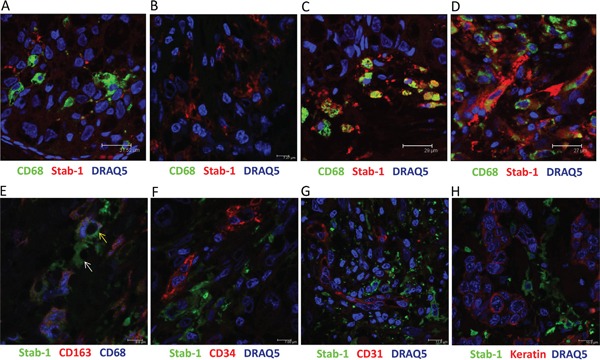
Heterogeneity of stabilin-1 expression by TAM in human breast cancer Immunofluorescent staining for co-expression of stabilin-1 with other cellular markers was performed on human breast cancer biopsies and analysed by confocal microscopy. Panel **A.** shows the area with low stabilin-1 expression on CD68high TAM; panel **B.** shows region with stabilin-1 expression on CD68low/negative cells; panel **C.** shows co-expression of stabilin-1 on CD68+ TAM; panel **D.** demonstrates the area with heterogeneous CD68 and stabilin-1 expression. Co-expression of stabilin-1, CD68, and CD163 is shown on panel **E.** yellow arrow indicates stabilin-1+CD163+CD68+ triple positive cell, white arrow indicates stabilin-1+CD163-CD68- cell; co-expression of stabilin-1 with CD34, CD31 and pan-cytokeratin is shown on panels **F**, **G** and **H.** respectively.

Crucial role of TAM in breast cancer progression was demonstrated in animal models of breast carcinoma, and positive association between TAM and disease progression was shown for human breast cancer [35–37]. M2-like phenotype was demonstrated for TAM subpopulations in animal breast cancer models [38, 39]. Since stabilin-1 was identified by us and others as a marker for alternative macrophage activation in rodents and human [1, 18], we first investigated its expression on TAM in tissue sections from patients with breast cancer using quantitative IHC and confocal microscopy. In parallel, staining for CD68 (pan-macrophage marker) and stabilin-1 ligand SPARC was performed. The expression of stabilin-1 was most pronounced on stage I disease, and in case of metastasizing primary tumors (stage IV). However, its expression decreased during TNM stages II and III (Figure 1A, 1B). Kruskal-Wallis analysis of stabilin-1 positivity showed significant difference in expression between TNM stages (p=0.0274) and Dunn's multiple comparisons test revealed significant difference between stage I and stage III disease. Interestingly, although there was slight trend for the decreased expression of CD68 on stages II and III disease the difference was not statistically significant (p=0.4512, Kruskal-Wallis test) (Figure 1C). The analysis of SPARC expression in analysed patient's cohort demonstrated that there was no association between SPARC levels and TNM stage (data not shown) indicating that SPARC is produced throughout all breast cancer clinical stages. However, strong trend for decreased SPARC expression in large tumors (stage T3) compared to stages T1 and T2 was observed and reached marginal significance (p=0.0696, Kruskal-Wallis test) suggesting inhibitory role of SPARC in tumor growth (Supplementary Figure 1). Immunofluorescent/confocal microscopy analysis of distribution of CD68 and stabilin-1 in human breast cancer samples of all stages demonstrated presence of three cell subpopulations: CD68^+^Stab-1^−^ (Figure 2A and Supplementary Figure 2A), CD68^−^Stab-1^+^ (Figure 2B and Supplementary Figure 2B), and CD68^+^Stab-1^+^ (Figure 2C Supplementary Figure 2C). These subpopulations could be found in the same tumor areas (Figure 2D), but frequently one of these subtypes was dominating in a given tumor region (Figure 2A-2C). Further phenotypic characterization of stabilin-1 positive cells using monocyte/macrophage marker CD163 revealed that CD68^+^Stab-1^+^ TAM co-expressed CD163 in certain tumor areas whereas CD68^−^Stab-1^+^ cells were typically negative for CD163 (Figure 2E and Supplementary Figure 3). Since stabilin-1 expression was found on intratumoral vessels in human urothelial bladder cancer and melanoma [18, 40] we have analysed co-expression of stabilin-1 with vascular markers CD34 and CD31 in human breast cancer. Stabilin-1 was not expressed by CD34+ blood vessels and majority of CD31+ vessels (Figure 2F, 2G and Supplementary Figure 4, 5A). However, in rare cases faint positive staining for stabilin-1 was observed on CD31+ structures suggesting intratumoral lymphatics as a possible source of stabilin-1 expression (Supplementary Figure 5B). In addition, co-staining with pan-cytokeratin antibodies excluded tumor cells as a source of stabilin-1 expression (Figure 2H and Supplementary Figure 6). Overall, our data indicated that stabilin-1 is expressed in human breast cancer with prevalent localization on TAM, and marks distinct subpopulation of cells in the tumor stroma with decreased or absent expression of CD68.

### Breast cancer growth is suppressed in stabilin-1 knockout mice

**Figure 3:**
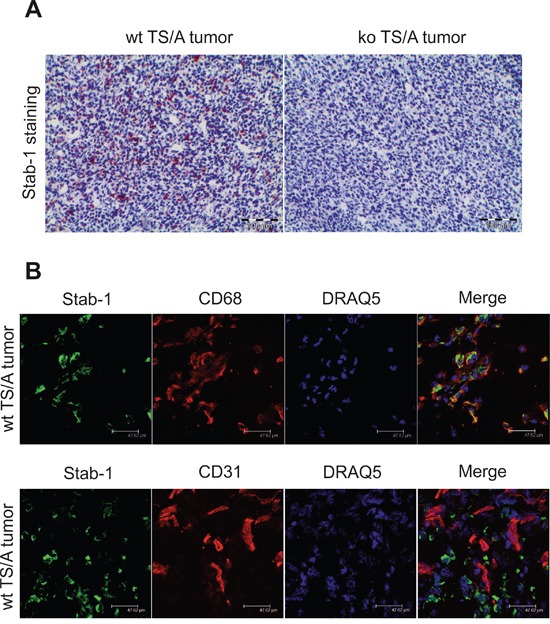
Stabilin-1 expression in TS/A mouse mammary adenocarcinoma tumor Wild type (wt) and stabilin-1 knockout (ko) mice were injected s.c. with 5×10^6^ TS/A tumor cells for 21 day. **A.** IHC analysis of stabilin-1 expression in TS/A tumors from wt and stabilin-1 ko mice. Representative images from 5 tumors are shown. **B.** Immunofluorescent/confocal microscopy analysis of CD68, CD31, and stabilin-1 expression in wt TS/A tumors 21 day after tumor cell inoculation. Representative images from 3 tumors are shown. Scale bars: 47, 62 μm.

Abundant expression of stabilin-1 in human breast cancer prompted us to examine its role in the regulation of primary breast tumor growth in a mouse model with genetic ablation of stabilin-1. Immunohistochemical analysis of TS/A tumors on day 21 after tumor cell inoculation revealed abundant stabilin-1 expression in wild type (wt) mice. As expected, expression of stabilin-1 in knockout (ko) mice was completely abrogated (Figure 3A). We and others previously demonstrated that stabilin-1 is expressed on two cell types: alternatively activated macrophages and non-continuous endothelial cells, including angiogenic vessels [1, 2]. Thus, we examined which cell type expresses stabilin-1 in TS/A tumor microenvironment. The data demonstrated that stabilin-1 is expressed on CD68+ TAM in TS/A tumor tissue but not on CD31+ micro-vessels (Figure 3B). Therefore, all subsequent studies of the impact of stabilin-1 on tumor growth concerned the number and function of TAM.

**Figure 4:**
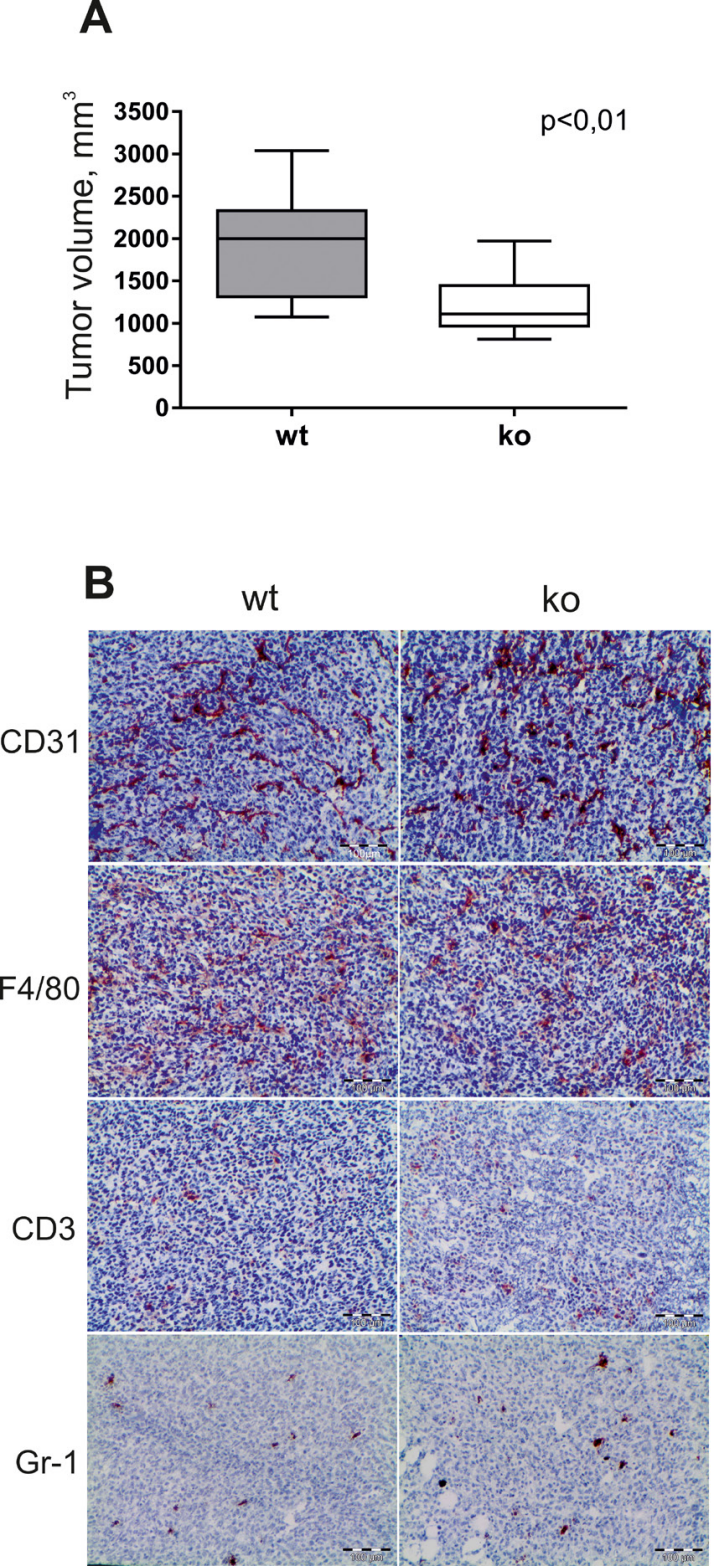
The effect of stabilin-1 on tumor growth and infiltration of TS/A tumors by immune cells Wild type (wt) and stabilin-1 knockout (ko) mice were injected s.c. with 5×10^6^ TS/A tumor cells. **A.** Tumor volume (mm^3^) in wt (n=12) and stabilin-1 ko (n=12) mice was measured 21 days after tumor cell inoculation. The data are presented as box plots with medians indicated, **p<0.01, Mann-Whitney U test. **B.** IHC analysis of TS/A tumors for the expression of CD31 (vascular marker), F4/80 (macrophage marker), CD3 (T cell marker) and Gr-1 (neutrophil marker) on day 21 after tumor cell inoculation. Representative images from 5 tumors are shown. Scale bars: 100 μm.

In order to examine the effect of stabilin-1 on tumor growth, Balb/c wt and stabilin-1 ko mice were injected subcutaneously with 5×10^6^ TS/A tumor cells. We found that 21 days post-inoculation tumor size was decreased by 36% in stabilin-1 ko mice compared to wt mice. The difference in tumor size was statistically significant (p<0.01; Mann-Whitney U test) (Figure 4A).

Potential effect of stabilin-1 knockout on infiltration of immune cells and angiogenesis was examined on day 21 of tumor growth. CD68+ and F/80+ macrophages were dominant immune cell type inside tumor mass (Figure 4B and data not shown). Single infiltrating CD3+ T cells and Gr-1+ neutrophils were found in tumors in both wt and stabilin-1 ko mice, and no difference was observed for both cell types as well as for the density of CD31+ micro-vessels (Figure 4B). We conclude that the absence of stabilin-1 in ko mice does not change the abundance of TAM and tumor vascularization. Therefore, we next asked whether new functions of stabilin-1 ko TAM might attenuate tumor growth in the TS/A tumor model.

### Endocytic clearance and SPARC uptake are affected in stabilin-1 knockout TAM

**Figure 5:**
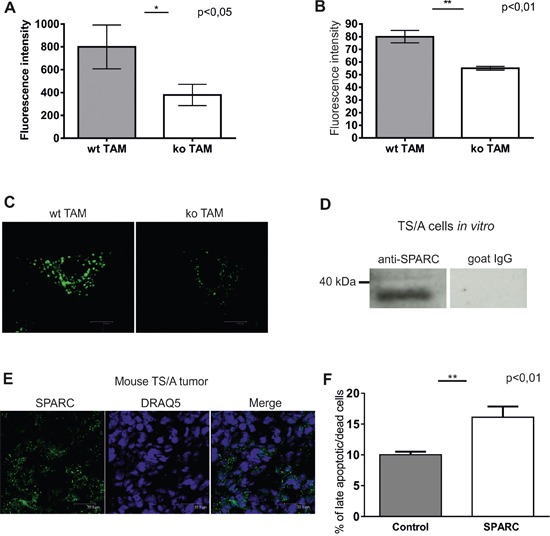
Endocytic clearance function is impaired in stabilin-1 knockout TAM TAM from wt and stabilin-1 ko animals were cultured overnight (15 h) followed by addition of acLDL-Alexa488 **A.** and SPARC-FITC **B.** at a final concentration of 5 μg/ml and 10 μg/ml respectively, and incubated for 30 min at 37°C. Quantification of surface bound/internalized ligands was performed using BD FACS Canto II flow cytometer. The data of one representative experiment out of three are presented as mean ± SD, *p<0.05 (n=3 for wt and ko), **p<0.01 (n=8 for wt, n=7 for ko), Student's t-test. **C.** TAM from wt (n=3) and stabilin-1 ko (n=3) mice were cultured overnight (15 h) followed by addition of SPARC-FITC at a final concentration of 10 μg/ml, and incubated for 30 min at 37°C. Cells were fixed in PFA and subjected to confocal microscopy analysis. Representative images of single cells without nuclear staining are shown. Scale bars: 8,86 μm (left panel) and 8,22 μm (right panel). **D.** TS/A cells were cultured *in vitro* and analyzed for SPARC expression using Western Blotting. Normal goat IgG was used as a negative control. **E.** TS/A tumor sections from 3 wt mice (21 days post-injection) were immunofluorescently stained using goat anti-mouse SPARC antibodies and scanned using Leica TCS SP2 confocal microscope. Representative images are shown. Scale bars: 22,5 μm. **F.** TS/A cells were cultured without stimulation or in the presence of recombinant SPARC (10 μg/ml) for 48h followed by assessment of percentage of late apoptotic/dead cells (SYTOX green+ and SYTOX Green+/annexin V+ cells) by flow cytometry. The data are mean ± SD of triplicates for one out of two representative experiments, **p<0.01, Student's t-test.

Stabilin-1 was previously identified by us as an endocytic receptor for multiple extracellular ligands [1]. Among known stabilin-1 ligands an extracellular regulator of tissue turnover SPARC is expressed in breast cancer and involved in the control of breast cancer growth [12, 41]. Since stabilin-1 mediated uptake of such ligands may limit their availability in the tumor microenvironment we have examined whether clearance function is affected in stabilin-1 ko TAM using common stabilin-1 ligand acetylated low density lipoprotein (acLDL) and protein ligand SPARC [6, 42]. Indeed, endocytic uptake of both ligands was significantly inhibited in stabilin-1 ko TAM isolated from TS/A tumors. Decrease in cell surface binding/internalization reached appr. 50% for acLDL and 30% for SPARC as detected by flow cytometry (Figure 5A, 5B). Since flow cytometry detects both surface-bound and internalized ligands we performed confocal microscopy experiment in order to demonstrate whether endocytosis of SPARC is affected in stabilin-1 ko TAM. Indeed, knockout of stabilin-1 resulted in evident abrogation of SPARC internalization and its transport to the endocytic pathway (Figure 5C). Nevertheless, some SPARC was found in small vesicles of stabilin-1 ko TAM suggesting involvement of pinocytosis in SPARC uptake by TAM. We have further demonstrated the expression of endogenous SPARC in TS/A cells cultured *in vitro* using Western blotting and in TS/A tumor tissues using immunofluorescent staining and confocal microscopy (Figure 5D, 5E). Comparable levels of SPARC expression were detected in wt and stabilin-1 ko tumors suggesting involvement of SPARC in the regulation of TS/A tumor growth in both mouse strains (Supplementary Figure 7). Since SPARC was reported to directly inhibit tumor cell proliferation and induce tumor cell apoptosis we tested both of these effects of recombinant SPARC on TS/A cells *in vitro*. Although extracellular SPARC did not influence proliferation of TS/A cells it significantly increased the percentage of late apoptotic/dead TS/A cells (Figure 5F, Supplementary Figure 8 and data not shown). Overall, our data indicate that stabilin-1 is involved in the clearance and sequestration of tumor growth modulating factors such as SPARC from extracellular space.

### Stabilin-1 does not induce transcriptional changes in TAM

**Figure 6:**
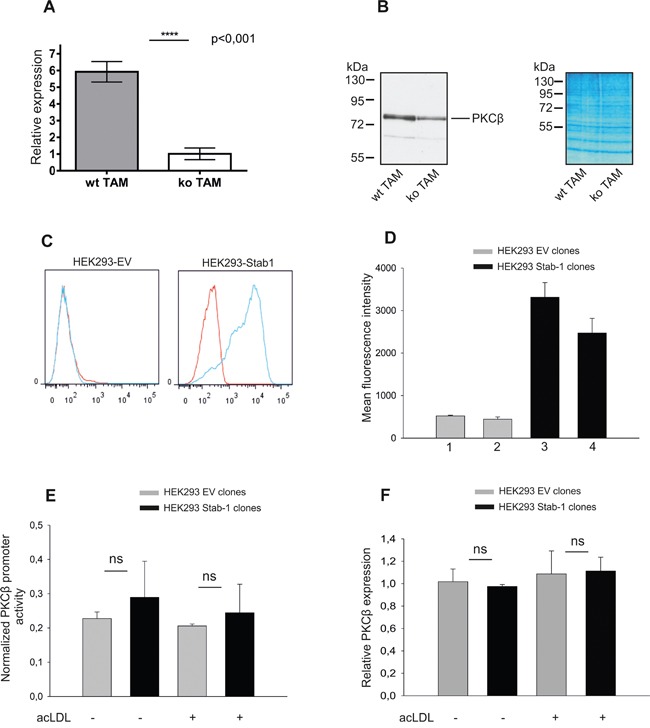
Assessment of stabilin-1 role in the activation of PKCβ gene expression **A.** TAM were isolated from wt (n=6) and stabilin-1 ko (n=6) mice and analyzed for PKCβ gene expression on mRNA level using Real-time PCR. Data are mean ± SD for one out of two experiments, **** p<0.0001, Student's t-test. **B.** TAM were isolated from wt (n=5) and stabilin-1 ko (n=5) mice and analyzed for PKCβ protein expression by Western blotting. Representative image and protein loading controls (right panel) are shown. **C.** The expression of stabilin-1 in HEK293 cells transfected with empty vector (HEK293-EV) and full-length stabilin-1 (HEK293-Stab1) was demonstrated by flow cytometry. Red histograms indicate isotype controls. **D.** The endocytosis of acLDL-Alexa488 by two HEK293-EV clones (1,2) and two HEK293-Stab1 clones (3,4) is presented. Data are mean ± SD of triplicates. **E.** HEK293-EV (n=2) or HEK293-Stab1 (n=2) clones were transfected with luciferase reporter construct containing human PKCβ promoter and stimulated with acLDL (5μg/ml) or left untreated. Luciferase activity was measured 48h after acLDL stimulation. The experiment was repeated 3 times. Data are mean ± SD; ns - not significant. **F.** HEK293 clones transfected with EV (n=3) or stabilin-1 (n=3) were stimulated with acLDL (5μg/ml) for 48h or left untreated and assessed for expression of endogenous PKCβ by Real-time PCR. Data expressed as mean ± SD, ns-not significant.

Clearance of endocytic ligands by scavenger receptors can be a silent event or can change the transcriptional profile of cells [43]. We next examined whether the absence of stabilin-1 affects expression profile of TAM using Affymetrix microarray assay. In order to identify differences in expression profile of native TAM exposed to ligands in tumor environment, freshly isolated TAM were used to extract RNA. We found that the absence of stabilin-1 expression in macrophages did not result in extensive changes in macrophage transcriptional program (Supplementary Figure 9). Real-time PCR analysis using independent cDNA samples from wt and stabilin-1 ko TAM did not detect statistically significant difference in expression of *lyve-1, fpr1, rps4y2, chek2, myoz1, plau, ptgr2, nt5e, il7r, tkt, sdc1*, and *retnla* genes (Supplementary Figure 10 and data not shown). However, the analysis revealed statistically significant reduction in expression of PKCβ in stabilin-1 ko TAM (Figure 6A) that was further confirmed by Western blotting (Figure 6B). These data suggested stabilin-1 involvement in the activation of PKCβ gene expression.

Next, we examined whether stabilin-1 mediates silent ligand clearance or can link clearance function with transcriptional changes. PKCβ gene, significantly affected by stabilin-1 knockout, was selected as a potential stabilin-1 responder gene. The effect of stabilin-1 on PKCβ expression was examined in HEK293 cells stably expressing functionally active human stabilin-1 (Figure 6C, 6D). However, presence of stabilin-1 in HEK293 cells neither induced the activity of transiently transfected PKCβ promoter nor expression of endogenous PKCβ (Figure 6E, 6F). Moreover, stimulation of stabilin-1 endocytic function using its potent endocytic ligand acLDL [6] did not result in the activation of PKCβ promoter (Figure 6E, 6F; acLDL+). These data indicated that stabilin-1 is rather mediating silent ligand clearance, and reduction of PKCβ in the stabilin-1 ko TAM is a developmental consequence of stabilin-1 absence not directly linked to stabilin-1 activity in tumors.

## DISCUSSION

In the current study we demonstrate the expression of scavenger receptor stabilin-1 on TAM in human breast cancer and reveal its tumor-promoting role using stabilin-1 ko breast cancer mouse model. Of note, expression of stabilin-1 in human breast cancer was most intensive in stage I and IV disease suggesting its crucial role for early primary tumor growth and progression. Using gene expression analysis and functional tests on purified TAM we revealed that increased tumor growth in wt mice was accompanied by enhanced endocytic clearance function of TAM which was characterized by enforced intracellular uptake of tumor growth inhibiting factor SPARC. However, stabilin-1 expression on TAM did not result in extensive transcriptional changes suggesting its role in silent clearance of extracellular ligands. Since some of these ligands (such as SPARC) are involved in the control of tumor progression, the regulation of their extracellular concentration by stabilin-1 can result in microenvironmental changes affecting tumor growth. In this study we have demonstrated that stabilin-1 endocytic ligand SPARC was indeed involved in induction of tumor cell death although its effect was moderate. We cannot exclude that due to promiscuous ligand-binding ability of stabilin-1 uptake of unidentified tumor-regulating ligands in addition to SPARC may contribute to overall tumor promoting effect of stabilin-1.

Nowadays, TAM are recognized as crucial cellular components involved in the regulation of tumor growth. Their tumor-supportive role in breast cancer is widely described [23, 24, 35, 37]. TAM are known to enhance breast tumor growth through production of growth factors, stimulation of angiogenesis, and suppression of adaptive immune responses [44–46]. Their phenotype is shaped by tumor microenvironment towards immunosuppressive state associated with increased production of tumor growth-promoting cytokines, decreased antigen-presenting properties, and elevated expression of scavenger receptors (SR) including SR-A, CD206, CD163, and stabilin-1 [31–33, 39, 47, 48]. Although the association of SR expression with poor outcome in several types of cancer was found their functional role in the modulation of tumor growth is obscure. Neyen et al recently demonstrated that SR-A contributed to tumor progression by interaction with unknown tumor cell-released ligand [49]. In other study SR-A expression on antigen-presenting cells was shown to dampen anti-tumor adaptive immune responses and decrease production of pro-inflammatory cytokines upon LPS stimulation [50]. However, in both studies SR-A ligands were not identified. The upregulation of stabilin-1 expression on TAM in different types of mouse and human tumors was previously described by us and other groups [18, 20–22]. We have also demonstrated that stabilin-1 is responsible for uptake of several extracellular ligands including modified low-density lipoproteins, apoptotic bodies, SPARC, TGFβ superfamily factor GDF15, and its expression is associated with alternatively activated macrophage phenotype [1, 3, 4, 6, 42]. Recently, Karikoski and colleagues demonstrated tumor-promoting role of stabilin-1 in mouse models of B16 melanoma and EL-4 lymphoma [22]. In agreement with this data, our results reveal tumor-supporting role of stabilin-1 in breast cancer mouse model. However, in contrast to the data by Karikoski et al, we did not observe expression of stabilin-1 on tumor vasculature in our mouse model. We cannot exclude that this phenomenon is mediated by differential activation of endothelium in melanoma and breast cancer microenvironments or is specific for TS/A tumor model. Moreover, the absence of stabilin-1 in our model did not affect macrophage infiltration into the tumor mass. The results of our study revealed that the clearance function of TAM in stabilin-1 ko mice was decreased and accompanied reduced tumor growth *in vivo*. We have proposed enhanced clearance of SPARC in wild type mice as a reason of increased tumor growth since tumor-inhibiting effect of extracellular SPARC in several types of cancer including breast cancer was widely demonstrated *in vitro* and *in vivo* [8, 10, 12, 14–16, 51–53]. Attenuating effect of SPARC on tumor growth was attributed to its direct cytostatic effect on tumor cells, as well as induction of tumor cell apoptosis and its regulatory effect on tumor ECM deposition. In our study we did not find anti-proliferative effect of SPARC on TS/A tumor cells. However, SPARC clearly enhanced death of TS/A cells supporting its role as a suppressor of breast cancer. This effect also corresponded to the identified by us reverse correlation of SPARC levels with tumor size (T-stage of breast cancer).

As a significant output, our study suggests the importance of stabilin-1-mediated silent ligand clearance in the modulation of tumor microenvironment as opposed to active clearance associated with changes in macrophage phenotype. For example, several studies indicate that uptake of apoptotic tumor cells by scavenger receptors on macrophages is accompanied by changes in macrophage expression and secretion profile [54–56]. In contrast, we demonstrate that expression of stabilin-1 on TAM directly isolated from breast tumor microenvironment is not accompanied by major changes in expression profile as assessed by gene microarray analysis. These data are in agreement with the fact that potential signaling motifs are absent in the cytoplasmic tail of stabilin-1 [1, 57]. In our study differential expression of PKCβ was the only significant difference between wt and stabilin-1 ko TAM validated by Real-time PCR analysis. However, we found that stabilin-1 is not involved in the regulation of PKCβ gene expression either on endogenous level or during transient transfection of PKCβ promoter-bearing construct. Furthermore, induction of stabilin-1 endocytosis did not result in changes of PKCβ promoter activation. In addition, we found that stabilin-1 ko tumor-bearing BALB/c mice have reduced expression of PKCβ not only in TAM but also in peritoneal macrophages, as well as in liver and kidney. Bone marrow macrophages isolated from naïve stabilin-1 ko mice were also characterized by reduced expression of PKCβ (data not shown). This implies that reduced expression of PKCβ is an intrinsic property of stabilin-1 ko Balb/c mice which is likely formed early during development. However, in TAM PKCβ expression is not directly regulated by stabilin-1. Overall, we suggest that modulation of tumor growth by stabilin-1 is mediated through dynamic removal of SPARC and presumably other unidentified tumor-regulating factors from extracellular space. The fact that endocytic activity of stabilin-1 is not coupled to the activation of transcription makes stabilin-1 an attractive molecular target on TAM for potential delivery of therapeutic drugs to inhibit pro-tumorigenic TAM activities.

In conclusion, our data demonstrate abundant expression of stabilin-1 in human breast cancer and reveal its importance for tumor growth in mouse breast cancer model. Based on our own results and studies from other groups we conclude that stabilin-1 may be involved in progression of many types of cancer and appears to be potential target for anti-tumor immunomodulatory therapy.

## MATERIALS AND METHODS

### Biopsy specimens

Breast cancer biopsy specimens of 31 patients seen at the University Medical Center Mannheim were used in the study. All studies were approved by the ethics and review committee of Medical Faculty Mannheim, University of Heidelberg (approval number: 2015-807R-MA).

### Animal tumor model

BALB/c wild type female mice were purchased from Janvier. Stabilin-1^−/−^ mice with BALB/c genetic background were generated in our facilities as described [3]. All mice were housed under specific pathogen-free conditions at the animal facility in Mannheim. All animal experiments including gene manipulations were approved by the Medical Ethics Committee II of the Medical Faculty Mannheim of the University of Heidelberg and filed with local authorities (Regierungspräsidium Karlsruhe). The BALB/c mammary TS/A adenocarcinoma cell line was kindly provided by Dr. Patrizia Nanni (Istituto di Cancerologia, Università di Bologna, Bologna, Italy) and cultured in RPMI+GlutaMAX medium (Gibco) supplemented with 10% FCS (Biochrom) and 100 μg/ml penicillin/streptomycin. Age-matched BALB/c wild type and stabilin-1^−/−^female mice of 8-14 weeks old were injected subcutaneously in the right flank with 5×10^6^ TS/A cells in PBS. Tumor volume (in mm^3^) was calculated using following formula: *Tumor volume = 0,52 × length × width × thickness*.

### Isolation of tumor-associated macrophages (TAM)

TS/A tumors were harvested 21 days after tumor cell inoculation and treated with 190 U/ml collagenase IV (Genaxxon) and 500 U/ml DNAseI (Roche) in OPTI-MEM medium (Gibco) for 1,5 h at 37°C. Released cells were passed through 100 μm cell strainer (BD Falcon) and subjected to positive selection using CD11b (human and mouse) magnetic beads (Miltenyi Biotec). CD11b^+^ cells were allowed to adhere on plastic for 30 min at 37°C, 5% CO2 in DMEM+GlutaMAX medium supplemented with 10% FCS and 100 μg/ml penicillin/streptomycin followed by removal of non-adherent cells. TAM purity of 92-95% was confirmed by flow cytometry using anti-CD11b (eBioscience) and anti-CD68 (AbD Serotec) antibodies. Typically, 1-3×10^6^ of TAM were isolated from single tumor.

### RNA isolation, microarray analysis and real-time PCR

RNA was isolated using the RNeasy midi kit (Qiagen, Hilden, Germany). Synthesis of cDNA was performed using Superscript II reverse transcriptase (Invitrogen). Gene expression profiling was performed using arrays of Mouse430_2 -type from Affymetrix (Santa Clara, CA, USA). cDNA and cRNA synthesis and hybridization to arrays were performed according to the recommendations of the manufacturer. A Custom CDF Version 12 with Entrez based gene definitions was used to annotate the arrays. The Raw fluorescence intensity values were normalized applying quantile normalization. Differential gene expression was analysed based on One-Way ANOVA, using a commercial software package SAS JMP7 Genomics, version 4, from SAS (SAS Institute, Cary, NC, USA). A false positive rate of a=0.05 with FDR correction was taken as the level of significance. The raw and normalized data were deposited in the Gene Expression Omnibus database (submission number GSE55595). Comparative analysis of gene expression was performed using Taqman Real-time PCR. Human PKCβ was amplified using predesigned TaqMan PKCβ assay (Hs00176998_m1, Life technologies), mouse PKCβ was amplified using following primers: TGCTGCATAATCGAGACACGC (forward), TGGAGGGTTGGGGTAAAGATGG (reverse), AGACCCCCACGGGAGAGAAAATGCTTGCTT (probe). Stratagene Mx3005 instrument was used for amplification. Predesigned TaqMan assays for *chek2, fpr1, il7r, lyve-1, nt5e, ptgr2, rps4y2, sdc1*, and *tkt* genes were purchased from Life technologies.

### Stabilin-1 expressing cell lines

HEK293 cells expressing full-length human stabilin-1 were generated in our facilities using pLP-IRESneo vector (Clontech). Positive clones were selected in DMEM+GlutaMAX medium (Gibco) supplemented with 10% FCS, 100 μg/ml penicillin/streptomycin and 500 μg/ml G418 (Life Technologies). Intracellular expression of stabilin-1 in stable clones was examined by flow cytometry using rabbit anti-stabilin-1 (clone RS1, self-produced [19] antibodies followed by staining with Alexa 488-conjugated a-rabbit ab (Dianova).

### Luciferase reporter assay

The pGL3 luciferase reporter construct containing 500 bp 5′-flanking sequence of human PKCβ promoter was kindly provided by Dr. Alan P. Fields (Department of Cancer Biology, Mayo Clinic Comprehensive Cancer Center, Jacksonville, Florida). Promoter activity was assayed by transfection of 1 μg pGL3-PKCβ promoter construct into HEK293 cells stably expressing stabilin-1 or empty vector clones using FuGene HD transfection reagent (Roche) as described by manufacturer. In parallel, HEK293 cells were transfected with 1 μg pGL3 basic plasmid or 1 μg pGL3 control plasmid (both from Promega). All cells were co-transfected with 200 ng of pRL-SV40 vector expressing Renilla luciferase (Promega) as an internal control for transfection efficiency. Forty eight hours after transfection, firefly and *Renilla* luciferase activity was measured using Dual-Luciferase Reporter Assay System from Promega according to manufacturer instructions. Luciferase activity was measured using luminometer Tecan Infinite 200. Results are expressed as firefly luciferase activity normalized to Renilla luciferase and firefly luciferase activity of cells transfected with pGL3 control plasmid.

### Immunohistochemistry, immunofluorescence and confocal microscopy

Paraffin embedded, formalin fixed, human breast cancer tissue sections (1μm thickness) were incubated in HIER T-EDTA Buffer pH 9,0 (Zytomed) for stabilin-1 and CD68 detection, or HIER T-EDTA Buffer pH 8,0 (Zytomed) for SPARC detection for 15 minutes in water bath at 80°C. Following primary anti-human antibodies were used: mouse CD68 (Zytomed), goat SPARC, clone AF941, goat CD163, sheep CD34 (all from R&D systems), rabbit anti-stabilin-1, clone RS1 (self-produced), mouse CD31 (DAKO), and mouse pan-cytokeratin (DAKO, clone AE1/AE3). Secondary antibodies were donkey anti-mouse, mouse anti-goat (both from Dianova) and donkey anti-rabbit (Amersham) HRP conjugates. Sections were developed using AEC Chromogen Substrate (Dako Cytomation) and counterstained with Mayer's Haemalaun solution (Merck). Entire sections were scanned using Nikon Eclipse Ni-E microscope equipped with NIS Elements Imaging Software (both from Nikon, Japan). Staining intensity (positivity) of whole sections was analysed using Aperio ImageScope program (free from Aperio.com) and Positive Pixel Count Algorithm. Raw intensity values obtained after analysis were normalized and presented as a fold change. All double and triple immunofluorescent stainings were performed in HIER T-EDTA Buffer pH 9,0. Alexa488, Cy3, and Alexa647 secondary conjugates raised in donkey were from Dianova. DRAQ5 (Cell Signaling Technology) was used for nuclear staining. Mouse tumors were harvested on day 21 after tumor cell inoculation. Frozen tissue sections (7 μm) were fixed in acetone for 10 min and probed with following anti-mouse antibodies: rat CD68 (Acris), rat F4/80-biotin, clone BM8 (Acris), rabbit anti-stabilin-1, clone RS1, hamster CD3e (eBioscience) rat CD31 (Abd Serotec), rat Gr1, clone RB6-8C5 (Abcam), goat SPARC (R&D systems, AF942). Immunohistological staining was performed using DAKO EnVision™+ System, Peroxidase (AEC) (Dako Cytomation). Images were acquired using Leica DC500 digital camera. Immunofluorescent data were acquired using Leica TCS SP2 and Leica TCS SP8 laser scanning spectral confocal microscopes equipped with a 63×1.32 objective and Leica Confocal Software.

### Western blotting

Lysates of TS/A tumor cells or freshly isolated TAM were subjected to SDS PAGE and transferred to nitrocellulose membranes (Protran). Following antibodies were used: goat anti-mouse SPARC (R&D systems, AF942), rabbit anti-mouse PKCβI, clone C16 or normal goat IgG (both Santa Cruz Biotechnologies). Secondary antibodies were HRP-labeled mouse anti-goat IgG (Dianova) and donkey anti-rabbit IgG (Amersham Biosciences). The membrane was developed using Luminata Forte western HRP substrate (Merck, Millipore).

### Endocytosis assay

Recombinant human SPARC was produced in Sf9 cells as described [42], and labeled with FITC using FluoroTag FITC Conjugation Kit (Sigma). acLDL-Alexa488 was purchased from Life Technologies. TAM were cultured for 15h as described above. Medium was changed for serum-free 30 min prior to addition of ligands. SPARC-FITC and acLDL-Alexa488 were added at a final concentration 10 μg/ml and 5 μg/ml respectively, and TAM were incubated for 30 min at 37°C. For HEK293 cells, acLDL-Alexa488 was added in final concentration of 2,5 μg/ml in serum-free DMEM medium for 30 min, 37°C. For microscopy analysis, cessation of endocytosis was achieved by immediate fixation in paraformaldehyde as described [57]. For flow cytometry, cells were placed on ice for 1 h and harvested using ice-cold PBS. Fluorescent signal was quantified using BD FACS Canto II flow cytometer (BD, Heidelberg, Germany). The data were analysed using WinMDI 2.8 and FlowJo 7.6.5 software.

### Tumor cell proliferation and apoptosis assay

TS/A cells (2×10^4^ per well) were seeded in 24 well plates, allowed to attach for 3h and stimulated with 10 μg/ml of recombinant SPARC protein (Peprotech) for 48h followed by trypsinization and assessment of proliferation using Click-iT® EdU Alexa Fluor® 488 Flow Cytometry Assay Kit (ThermoFisher Scientific) or apoptosis/cell death using Dead Cell Apoptosis Kit with Annexin V APC and SYTOX® Green (ThermoFisher Scientific) according to manufacturer instructions. Cell proliferation and apoptosis were analysed by flow cytometry using BD FACS Canto II flow cytometer.

### Statistical analysis

The difference in tumor growth between wt and stabilin-1 ko mice was analysed using Mann-Whitney U test and GraphPad Prism 6 software (GraphPad Soft Inc., La Jolla, CA). The association of stabilin-1, CD68 and SPARC expression with clinical stages was analysed using Kruskal-Wallis test. The rest of the data were analysed using two-tailed t test and are expressed as mean ± SD. Differences were considered significant at p<0.05 level.

## SUPPLEMENTARY FIGURES


