# Supplementary material for: Stabilin-1 is expressed in human breast cancer and supports tumor growth in mammary adenocarcinoma mouse model
Source: Oncotarget. 2016 Apr 20;7(21):31097–110. doi: 10.18632/oncotarget.8857 (PMC5058742; doi:10.18632/oncotarget.8857)
Supplement: Supplementary file 1 [file oncotarget-07-31097-s001.pdf]

SUPPLEMENTARY FIGURES

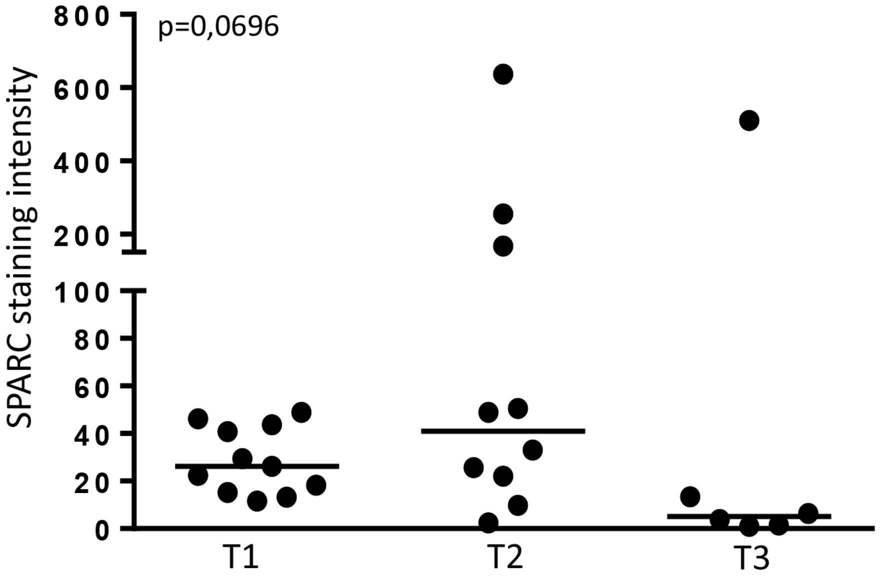

Supplementary Figure S1: Quantitative IHC analysis for the intensity of SPARC expression on stages T1, T2, and T3 of breast cancer (n=29). The data are presented as a fold change; p=0.0696, Kruskal-Wallis test.

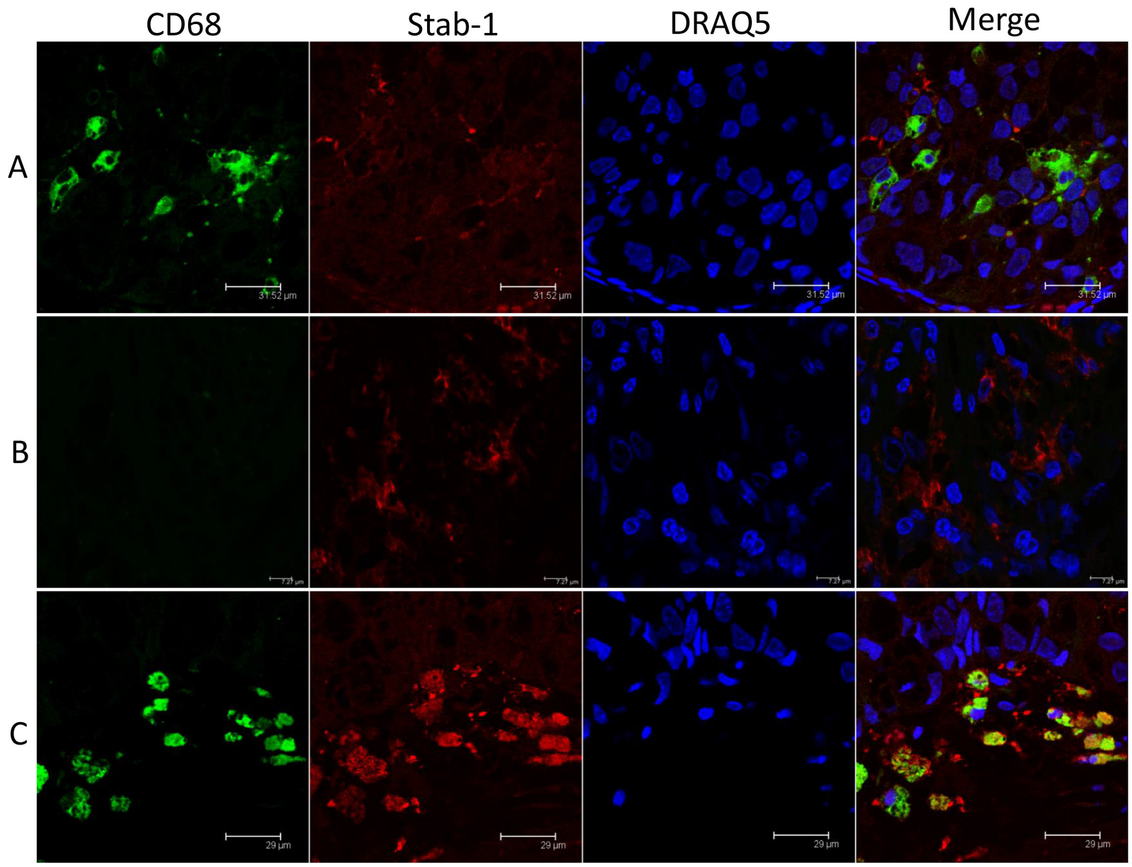

Supplementary Figure S2: Co-expression of stabilin-1 and CD68 in human breast cancer. Formalin-fixed paraffin-embedded sections were stained using mouse anti-CD68 and rabbit anti stabilin-1 (clone RS1) antibodies and analyzed using confocal microscopy. DRAQ5 was used to visualize nuclei. Representative images for CD68+stab-1- **A**. CD68-stab-1+ **B**. and CD68+stab-1+ **C**. cell subpopulations are shown.

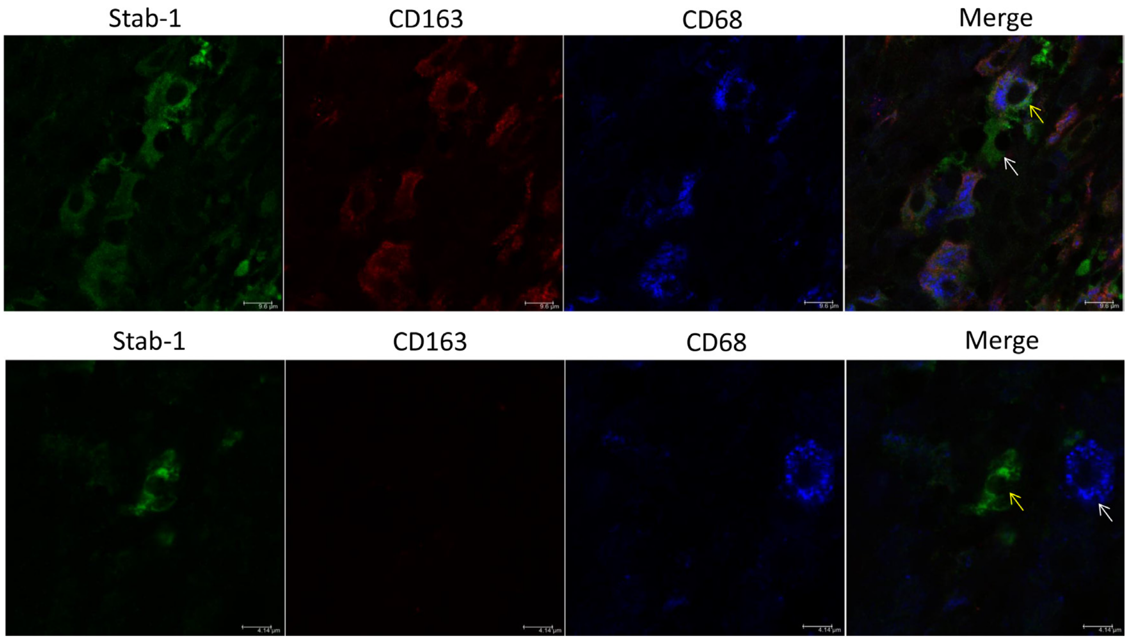

Supplementary Figure S3: Co-expression of stabilin-1 with CD163 and CD68 in human breast cancer. Formalin-fixed paraffin-embedded sections were stained using mouse anti-CD68, rabbit anti stabilin-1 (clone RS1), and goat anti CD163 antibodies followed by confocal microscopy analysis. Upper panel shows region with expression of all three markers. Yellow arrow indicates stab-1+CD163+CD68+ triple positive cell, white arrow indicates stab-1+CD163-CD68- cell. Lower panel shows examples of stab-1+CD163-CD68- cell (yellow arrow) and stab-1-CD163-CD68+ cell (white arrow).

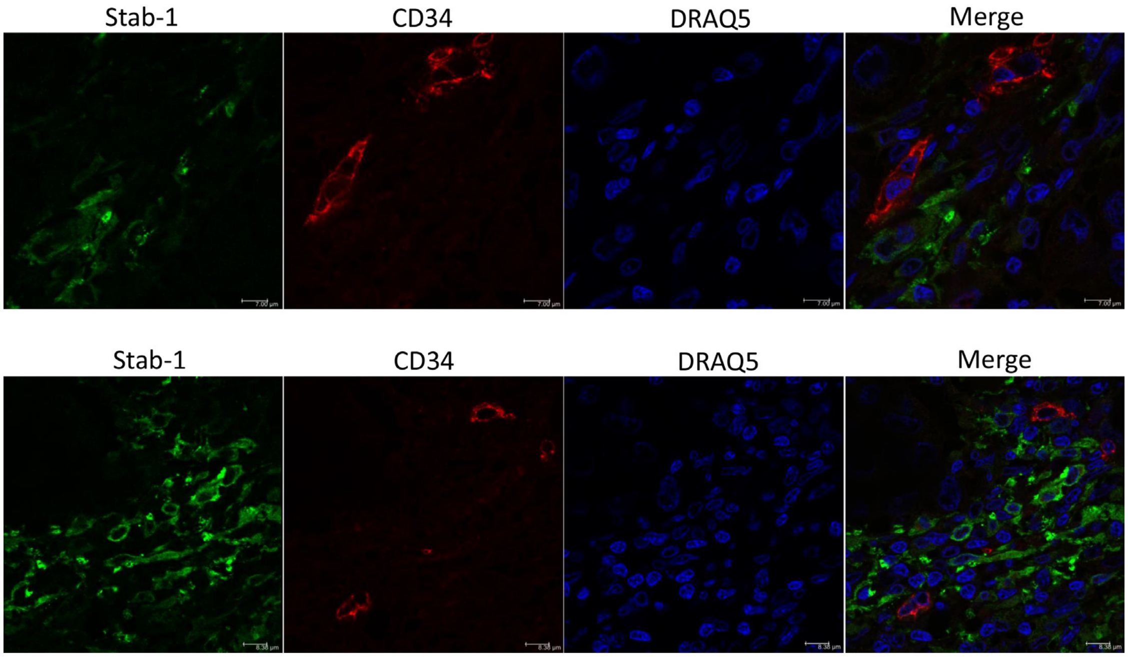

Supplementary Figure S4: Expression of stabilin-1 and CD34 in human breast cancer. Formalin-fixed paraffin-embedded sections were stained using sheep anti-CD34 and rabbit anti stabilin-1 (clone RS1) antibodies and analyzed using confocal microscopy. DRAQ5 was used to visualize nuclei. Representative images are shown.

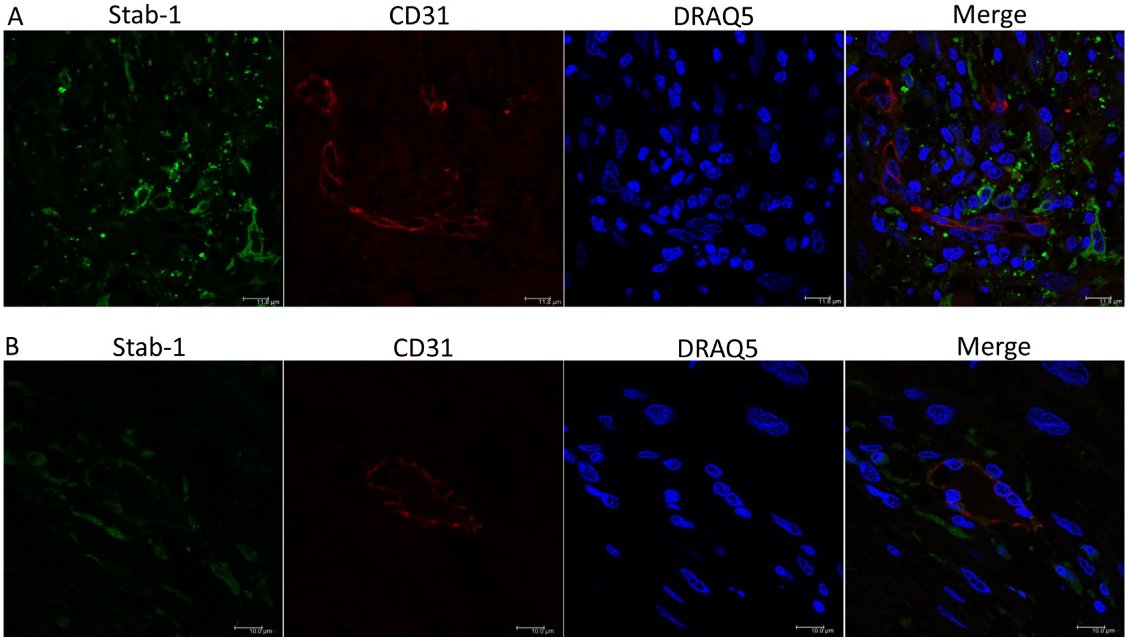

Supplementary Figure S5: Expression of stabilin-1 and CD31 in human breast cancer. Formalin-fixed paraffin-embedded sections were stained using mouse anti-CD31 and rabbit anti stabilin-1 (clone RS1) antibodies and analyzed using confocal microscopy. DRAQ5 was used to visualize nuclei. **A**. An example of CD31+stab-1- vessel is shown. **B**. An example of CD31+ vessel with dim expression of stabilin-1 is shown.

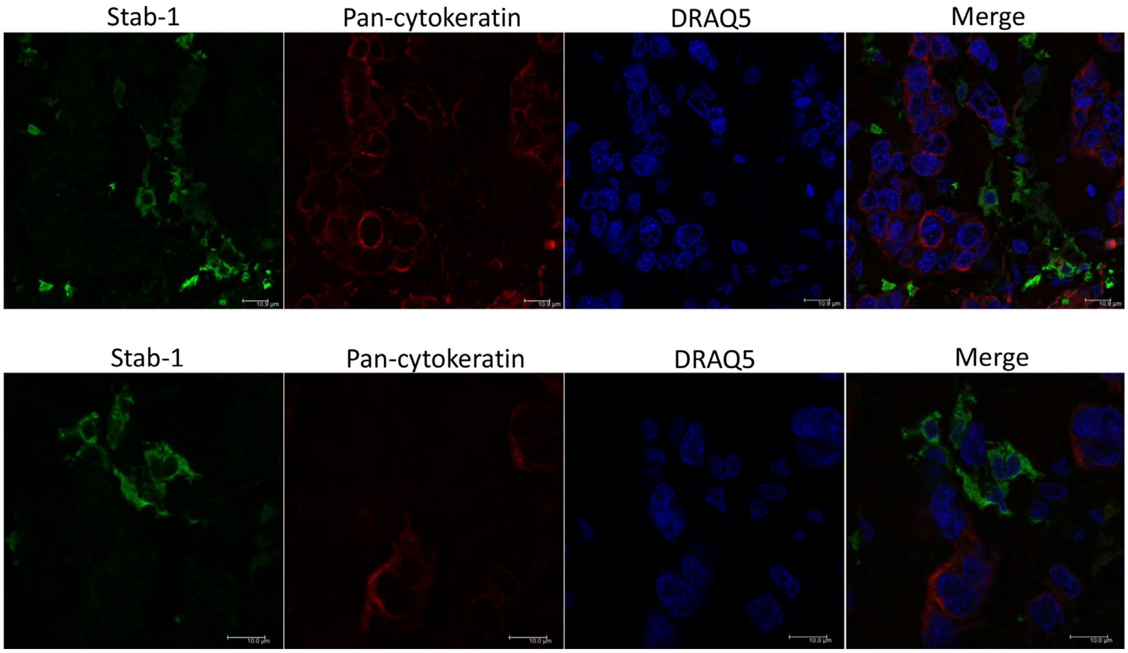

Supplementary Figure S6: Expression of stabilin-1 and pan-cytokeratin in human breast cancer. Formalin-fixed paraffin-embedded sections were stained using mouse anti pan-cytokeratin and rabbit anti stabilin-1 (clone RS1) antibodies and analyzed using confocal microscopy. DRAQ5 was used to visualize nuclei. Representative images are shown.

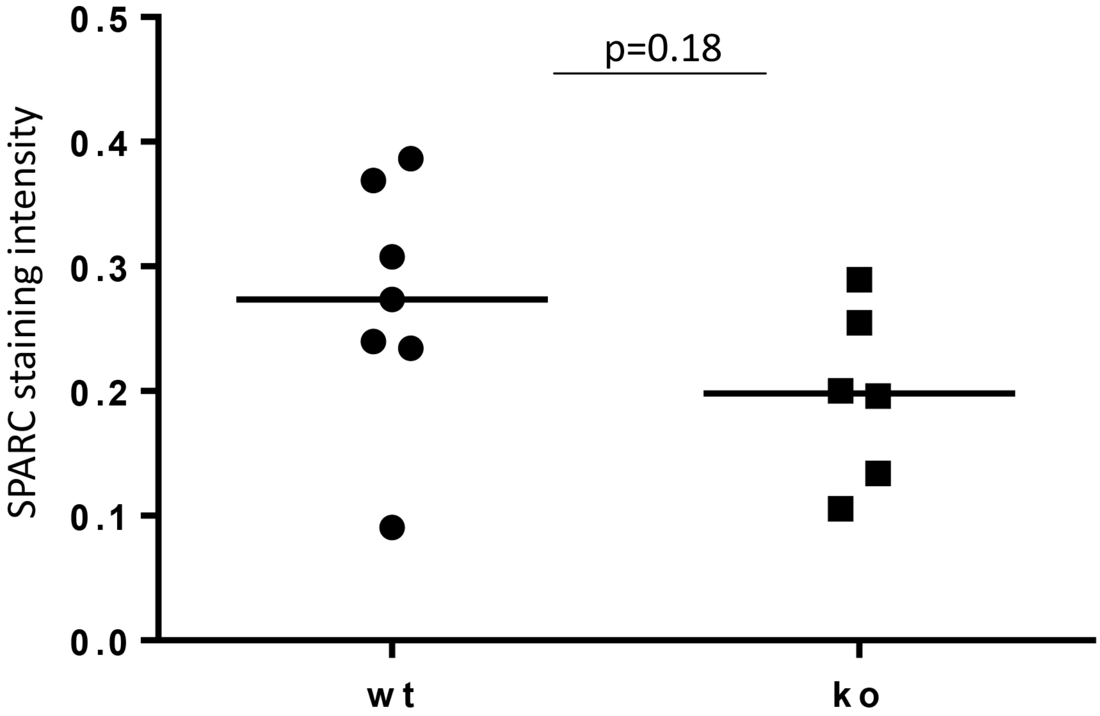

Supplementary Figure S7: Quantitative IHC analysis for the intensity of SPARC expression in TS/A tumors of wt (n=7) and stabilin-1 ko (n=6) mice. The intensity data are presented as a ratio positive pixels/total pixels; p=0.18, Mann-Whitney U test).

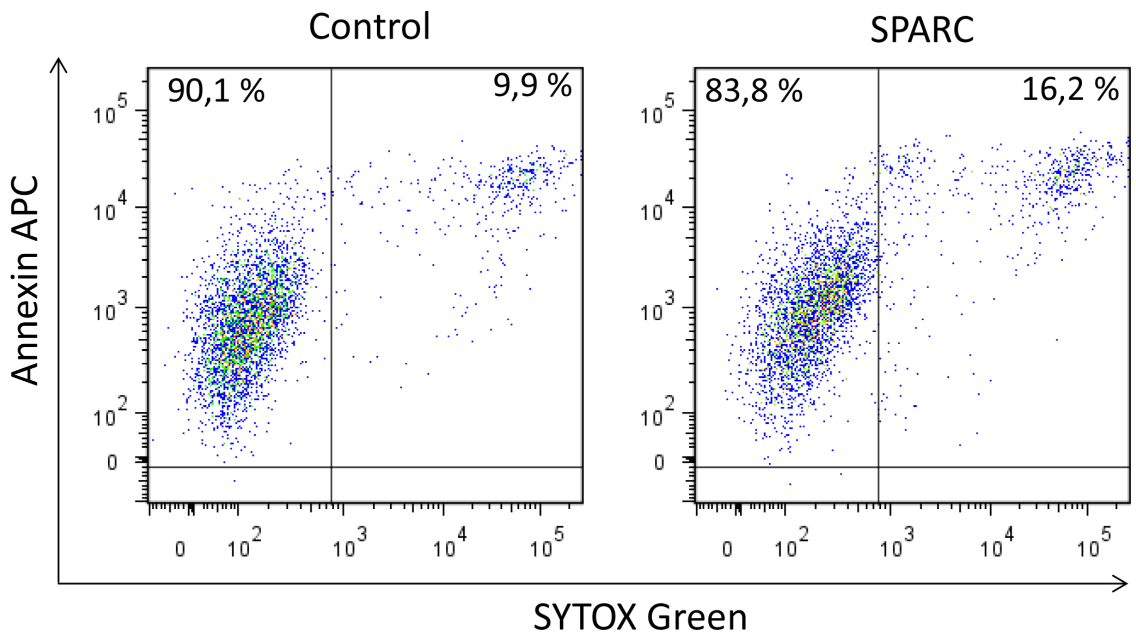

Supplementary Figure S8: The effect of SPARC on TS/A cell death and apoptosis *in vitro*. TS/A cells were cultured in the presence of 10 µg/ml of recombinant SPARC and the percentage of late apoptotic/dead cells (SYTOX Green+ and annexin V+SYTOX Green+) was assessed using flow cytometry. Representative dot plots from control and SPARC-treated cells are shown.

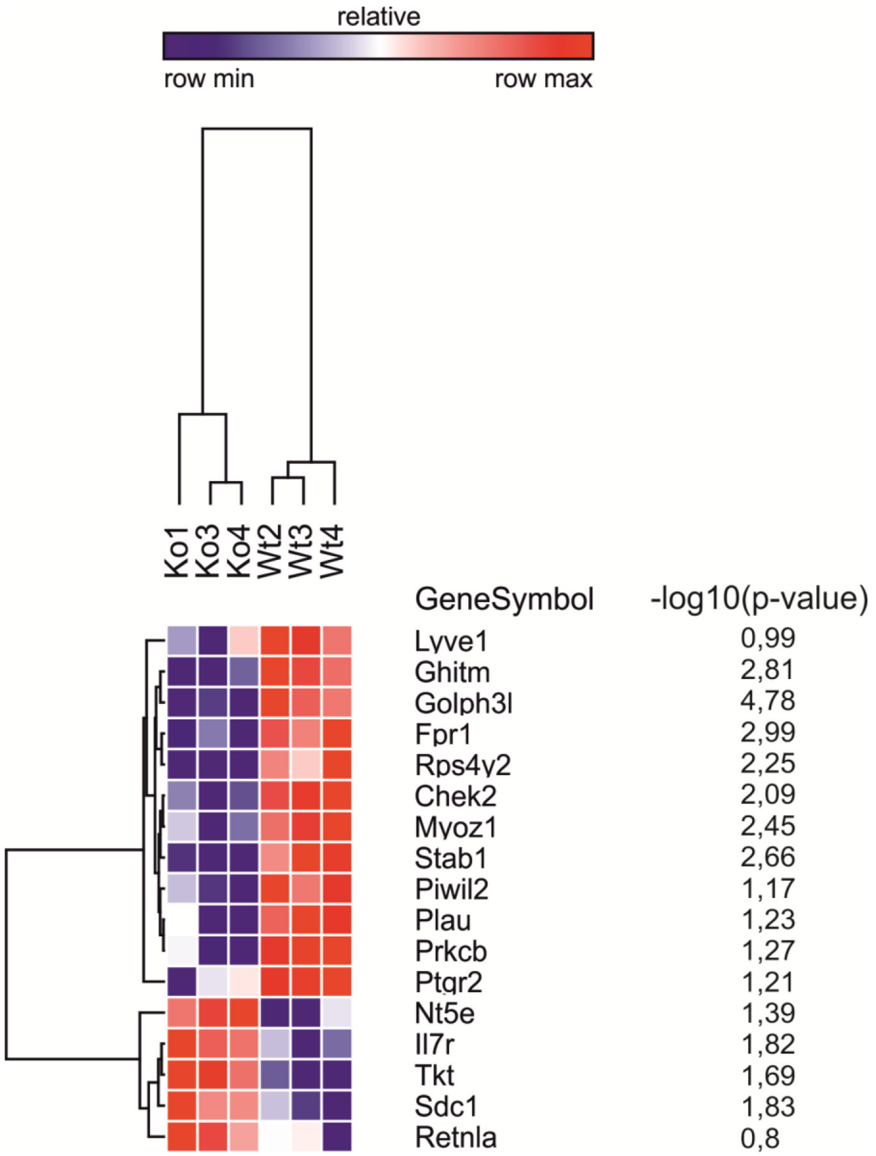

Supplementary Figure S9: Affymetrix gene microarray analysis of TAM. TAM were isolated from 3 wt and 3 stabilin-1 ko mice 21 days after TS/A cell injection and analyzed by Affymetrix gene chip microarrays. Heat-map of differentially expressed genes and corresponding p-values are presented.

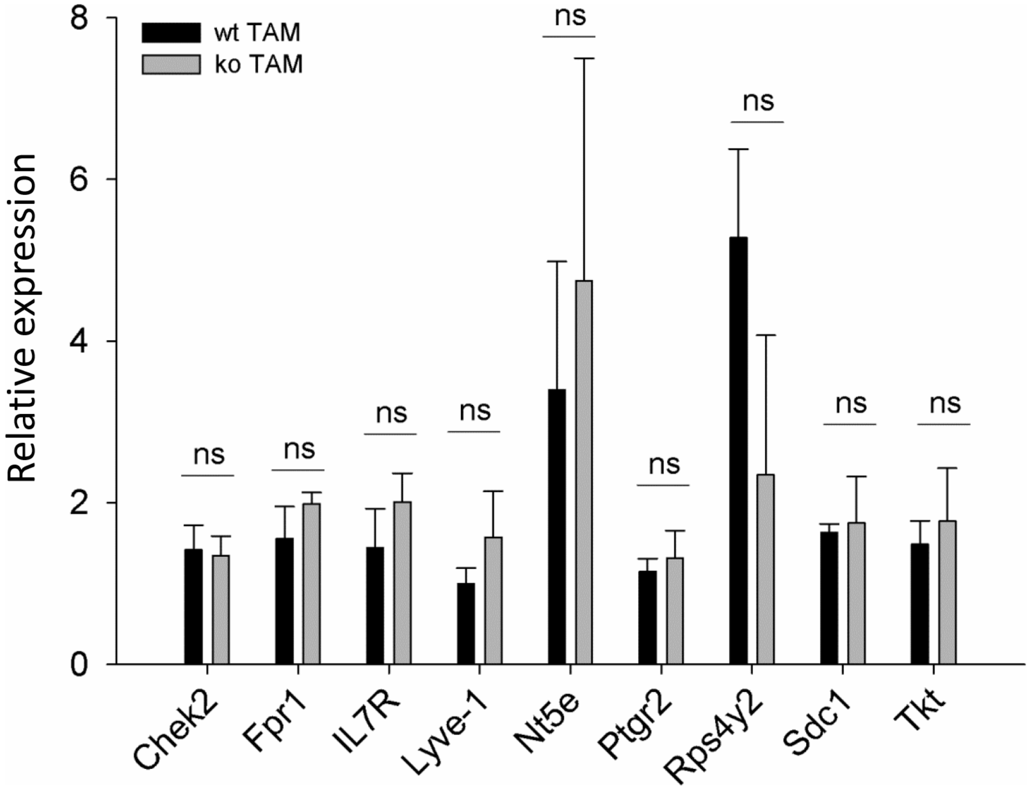

Supplementary Figure S10: Validation of microarray data by Real-time PCR. TAM were isolated from 3 wt and 3 stabilin-1 ko mice 21 days after TS/A cell injection and analyzed for the expression of *chek2, fpr1, il7r, lyve-1, nt5e, ptgr2, rps4y2, sdc1*, and *tkt* genes by Real-time PCR. Data are expressed as mean ± SD, ns – not significant, Student's t-test.
